# Effectiveness of Face Coverings in Mitigating the COVID-19 Pandemic in the United States

**DOI:** 10.3390/ijerph18073666

**Published:** 2021-04-01

**Authors:** Olukayode James Ayodeji, Seshadri Ramkumar

**Affiliations:** Department of Environmental Toxicology, The Institute of Environmental and Human Health, Texas Tech University, Lubbock, TX 79416, USA; James.Ayodeji@ttu.edu

**Keywords:** COVID-19, face covering, pandemic, SARS-CoV-2, state-wide mandate

## Abstract

The COVID-19 pandemic has been one of the biggest public health challenges of the 21st century. Many prevalent measures have been taken to prevent its spread and protect the public. However, the use of face coverings as an effective preventive measure remains contentious. The goal of the current study is to evaluate the effectiveness of face coverings as a protective measure. We examined the effectiveness of face coverings between 1 April and 31 December 2020. This was accomplished by analyzing trends of daily new COVID-19 cases, cumulative confirmed cases, and cases per 100,000 people in different U.S. states, including the District of Columbia. The results indicated a sharp change in trends after face covering mandates. For the 32 states with face covering mandates, 63% and 66% exhibited a downward trend in confirmed cases within 21 and 28 days of implementation, respectively. We estimated that face covering mandates in the 32 states prevented approximately 78,571 and 109,703 cases within 21- and 28-day periods post face covering mandate, respectively. A statistically significant (*p* = 0.001) negative correlation (−0.54) was observed between the rate of cases and days since the adoption of a face covering mandate. We concluded that the use of face coverings can provide necessary protection if they are properly used.

## 1. Introduction

The COVID-19 outbreak has spread to 224 countries and territories, infecting about 95 million people around the globe and resulting in over 2 million confirmed deaths [1]. The United States has the highest number of cases and positive case rate with over 27.2 million cumulative cases at 8218.9 reported cases per 100,000 people, as of 9 February 2021 [2,3]. As of the same date, the pandemic has caused over 467,312 fatalities in the United States [3]. As new evidence becomes available, knowledge of the spread of the virus continues to evolve. Current evidence suggests that it is mainly transmitted through ejected respiratory droplets (>5 μm), smaller aerosols (<5 μm), and direct contact with an infected person [4]. Infected droplets may be ejected by asymptomatic, symptomatic, and pre-symptomatic individuals [5,6,7] when they speak, sneeze, or cough [4]. If a healthy individual inhales suspended and/or traveling infected droplets and aerosols, they allow the virus to enter the respiratory system, resulting in infection. 

The mechanism of spread of severe acute respiratory syndrome coronavirus 2 (SARS-CoV-2) that causes COVID-19 is not fully understood yet, especially in terms of the contributions of environmental (physical and social) factors [8,9] and the relative contributions of airborne and direct contact transmission routes [10]. A recent analysis of the pandemic trends in three epicenters (i.e., Wuhan, China; Italy; and New York City) indicated that the dominant route of transmission is airborne, and face coverings significantly impacted the trend of the outbreak [10]. Face coverings have been recommended for use because they offer a physical barrier against the transmission of respiratory viruses [4,11]. With increasing evidence on the airborne transmission of the virus, wearing face coverings was one of the major CDC recommendations for reopening the United States after the COVID-19 pandemic lockdown [12]. 

Because SARS-CoV-2 is highly contagious, COVID-19 is an inherently social phenomenon, and limiting social contact is effective in containing it [8]. For this reason, most mitigating measures emphasized physical distancing such as lockdowns, quarantine, required minimum distance, isolation, stay-at-home orders, and shelter-in-place. At the onset of the COVID-19 outbreak, all states in the U.S., including the District of Columbia, made emergency declarations and 42 states issued mandatory stay-at-home orders between 1–31 March 2020 [13]. Similarly, most states prohibited or restricted non-essential services (46 states) including bar closures (all states) and dine-in services (49 states) [14]. The U.S. Centers for Disease Control and Prevention suggested maintaining at least 2 m (6 foot) from others to reduce the risk of infection from ejected and infected droplets [11]. Even before the enactment of these public measures for COVID-19, media and authorities encouraged people to voluntarily practice physical distancing behaviors to reduce the transmission of the virus [8]. 

Although physical distancing has been mostly accepted and practiced, the use of face coverings has not been fully embraced and it remains a highly debated issue [15,16,17]. The controversy about the use of face coverings during the COVID-19 pandemic originated from ideological differences, perception of risk, uncertainty, and lack of understanding of its effectiveness [15,18,19,20]. The prevailing view presupposes the variation in experimental results [19,21] and limited available evidence so far on how community use of face coverings would affect the spread of COVID-19 [22]. Moreover, debate also occurred among the research communities [19], political entities, and decision-makers [15]. The implementation of face covering measures varies greatly from one state to another [23] and there is an increasing divide between those who wear and who do not wear masks [15]. 

As SARS-CoV-2 is a novel virus, its mechanism of transmission and consequent intervention is unprecedented. Suitable methods to model the transmission and assess the effectiveness of intervention measures for the pandemic have not yet been fully developed [20], and available frameworks for its containment require scientific validation. Attempts have been made towards understanding the effectiveness of face coverings against the spread of COVID-19. However, analysis is often carried out using mathematical and empirical modeling approaches [18,22,24,25], simulation experiments [26,27], and participatory surveys [19,27,28]. To our best knowledge, no report has documented the effectiveness of face coverings using comprehensive data from almost all states in the U.S. and actual COVID-19 infection cases data between 1 April to 31 December 2020, in a single study. In the current study, actual confirmed cases, a longer timeframe, and up to 46 states were included in the analysis. We examined the effectiveness of face coverings as a mitigating measure in the U.S. during the COVID-19 pandemic. This objective was accomplished by analyzing the trends of daily new COVID-19 cases, cumulative confirmed cases, and infection cases per 100,000 people in different states including the District of Columbia. 

## 2. Results and Discussion 

### 2.1. Distinctive Change in Trend after Face Covering Mandate

**Figure 1:**
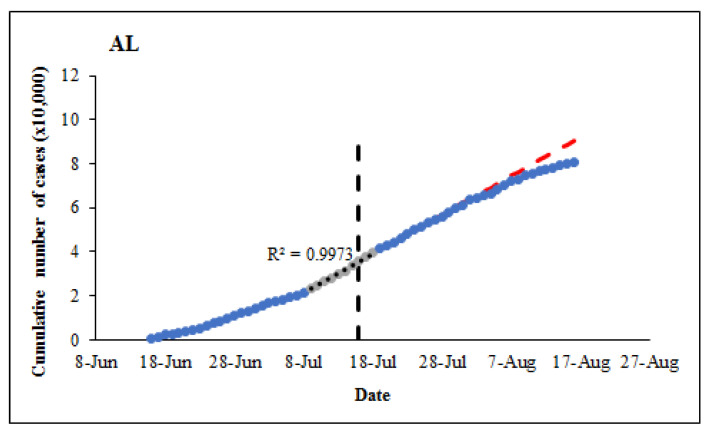
Cumulative number of COVID-19 cases in Alabama state between 16 June–19 August. Linear regression equation (R^2^ = 0.9973) was used to estimate the number of cases prevented by 21 days (5804) and 28 days (10,763), after the face covering (FC) mandate. The slope (d^−1^) indicates daily rate of change in confirmed cases.

A distinctive change in trend was observed in the number of daily new cases and total confirmed cases in most states after state-wide face covering (FC) mandates were enacted (Supplementary Figure S1). Depending on the state, this change coincides with and occurs a few days after FC was mandated. The effectiveness of FC use on daily new cases and cumulative confirmed cases was assessed by computing the difference between reported and projected numbers. This assessment was justified by the high coefficient of determination values (R^2^ ranged from 0.9774 to 0.9987) obtained for regression equations and by limiting the projection to 28 days post FC mandate. The prediction with regression equation indicated a reduction in daily and cumulative number of cases in 63% and 66% of the states, 21 and 28 days post FC mandate, respectively (Table 1). For instance, after the FC mandate in Alabama on 16 July 2020, it was estimated that 5804 cases were prevented within 21 days and the number almost doubled in another 7 days (Figure 1). After 21 and 28 days, the three largest differences were observed in New York (111,417 and 160,956 respectively), Iowa (49,750 and 69,448 respectively), and Massachusetts (23,816 and 35,104 respectively) (Table 1). 

It is important to understand the 1st-order processes for the COVID-19 pandemic, which includes the transmission route of the virus, intervention measures, and the interactions that exist between the above two factors [20]. In the current study, the key feature in the curves is a change in case trends after state-wide FC mandates (Supplementary Figure S1). This distinctive change represents a dynamic equilibrium that was attained between transmission and intervention after few days. Second-order factors that are likely to impact the COVID-19 infection trend to include an incubation period [29], which is the time from exposure to the time when symptoms are developed in a typical symptomatic situation. The incubation period of SARS-CoV-2 is estimated to be 5 days [29]. Therefore, in the study, we excluded data from the 7-day period post FC mandate, including the date of the mandate. 

A noticeable change was observed within a few days after the implementation of the FC measures in Connecticut, Delaware, Iowa, Louisiana, Maryland, Massachusetts, New Mexico, New York, North Dakota, Ohio, Wisconsin, and Wyoming (Supplementary Figure S1). It appeared that the effectiveness of state-wide mandates of FCs in public was observed within a few days in the majority of the states. Similar analysis indicated that FC is a determinant factor in changing the trend of infection cases during a pandemic [10]. Community-wide wearing of FCs in public is effective in preventing interhuman transmission [15,22,30], especially where significant distance between individuals cannot be achieved. In states with FC mandates included in the current study, it was estimated that at least 78,571 cases were prevented within 21 days after the mandate and at least 109,703 within 28 days. 

Differences in trend are still evident in the results (Table 1 and Supplementary Figure S1) among all the states that have FC mandates. The differences may be attributed to behavior and policy implementation that varies from one state or county, to another. For instance, comfort, socio-economic factors, cost of compliance, perception of risk, incentives, and social connectivity have been documented to impact mitigation compliance [31,32,33]. Similarly, implementation policies vary from county to county. For example, counties in Colorado, Kansas, and Ohio may elect to opt out of statewide FC orders if they meet certain benchmarks for declining caseloads, are deemed to be at low risk for transmission, or meet other public health criteria [23]. These may in part explain why 34% and 37% of the states with FC mandates do not exhibit a reduction in case trends by 28 and 21 days after the mandate, respectively.

### 2.2. Confirmed Infection Cases Rate

Case rates for states with and without FC mandates were compared during the two exponential increases in confirmed cases. Here, confirmed case rates were used for comparison to normalize the differences in population size. Seven out of 10 states (70%) with the lowest case rates by the end of the summer exponential increase in cases have FC mandates while all states (100%) with the lowest case rates by the end of the fall exponential increase have FC mandates (Table 2). On the other hand, 7 out of 10 states (70%) with the highest case rates by the end of summer did not have FC mandates. Eight out of 10 states with the highest number of case rates by the end of the fall exponential increase did not have effective FC mandates during the period. For instance, South Dakota, Nebraska, Tennessee, and Idaho did not have FC mandates while North Dakota (14 November), Iowa (14 November), Utah (9 November), and Wyoming (9 December) only recently mandated FC requirements (Table 3). 

Physical distancing and hand sanitizing help to prevent contact transmission of SARS-CoV-2 but not airborne transmission [20]. The use of FCs in combination with physical distancing provides maximum protection against both contact and airborne exposures [10]. It was reported that public use of FCs greatly reduced COVID-19 growth rates in U.S. states that enacted FC mandates [22]. The results observed in the current study also suggested that state-wide FC requirements provided an additional layer of mitigation to offer maximum protection against the virus. During the periods (summer and fall), lower case rates were noticeable in most states with FC mandates while higher rates were observed in most states without the mandate (Table 2 and Table 3). These lower case rates may be associated with compliance with mandates. Since COVID-19 is an inherently social phenomenon [8], it requires communal response [22]. By relying on what [34] was described as priming reasoning and encouragement by media, authorities, and peers, many people in the U.S. and around the globe voluntarily followed FC recommendations whether mandated or not. However, the results of lower case rates and trends in states where FC is mandated are evidence-based [20,22].

### 2.3. Change in Trends of Confirmed Infection Rate

It is important to determine whether the use of FCs had any impact during summer (20 June–22 September) and fall (22 September–21 December) when there was a sharp increase in the number of cases. We assessed the effects of FCs on infection cases per 100,000 people by comparing the slopes (rate of change in cases per day) of the linear regression during the summer and fall increase in daily new cases. Slopes for the entire data period (1 April–31 December) were also computed. A relationship between infection cases per 100,000 people (*y*-axis) and time (*x*-axis) was established. Here, since normalized cases per 100,000 people data were used for analysis, 46 states were included in the analysis. Thirty-five states with FC requirements and eleven states without the requirements were included in this analysis excluding states with a lower cumulative number of confirmed cases (HI, ME, NH, RI, and VT). Results are presented in Supplementary Table S2. Steep slopes denote increasing trends while smaller slopes indicate slowing trends. That is, the higher the slope, the higher the case rate and vice versa. The differences in trends between states with and without FC mandates were distinguishable. 

#### 2.3.1. Summer Exponential Increase in Confirmed Cases (20 June–22 September)

The linear regression equation slope ranged from 3.039 to 31.396. The 10 states with lower slopes were Connecticut (3.0394), New York (3.4079), New Jersey (4.2512), Massachusetts (4.6683), Pennsylvania (5.9357), Oregon (6.5497), Wyoming (6.7687), Colorado (6.774), Michigan (6.9972), and the District of Columbia (8.2233). Eight of these states (80%) with the lower slopes had FC mandates by 22 September 2020. The remaining two states (Michigan and Wyoming) later mandated FC by October 5 and December 9, respectively. The 10 states with higher slopes were Florida (31.396), Mississippi (28.606), Louisiana (28.59), Georgia (28.567), Alabama (27.364), Tennessee (25.286), Nevada (24.331), South Carolina (24.303), Arizona (23.349), and Texas (23.242). Six of these states (60%) did not have FC mandates by 22 September 2020.

#### 2.3.2. Fall Exponential Increase in Confirmed Cases (22 September–21 December)

Slopes ranged from 17.668 to 122.19. The 10 states with lower slopes were the District of Columbia (17.668), Oregon (19.036), Washington (20.046), Virginia (20.928), New York (21.314), Maryland (24.415), California (24.569), Florida (25.567), Georgia (25.815), and South Carolina (26.54). Seven of these states (70%) had a FC mandate by 21 December 2020. The 10 states with higher slopes were North Dakota (122.19), South Dakota (108.2), Wisconsin (81.488), Wyoming (81.135), Montana (76.922), Iowa (75.765), Nebraska (74.254), Utah (68.465), Minnesota (68.138), and Indiana (61.306). Two (South Dakota and Nebraska) of the 10 states did not have a FC mandate by 21 December 2020. North Dakota, Wyoming, Iowa, and Utah mandated FCs by 14 November, 9 December, 17 November, and 9 November, respectively. Essentially, six of these states (60%) did not have a FC requirement for most of the period.

The exponential periods observed in the current study coincided with summer vacations, onset of fall, thanksgiving holiday, and transitioning from summer to fall, among others. It was demonstrated that SARS-CoV-2 can persist significantly longer at lower temperatures than generally thought possible and remain infectious [35]. Increased human activities and drop in ambient temperature during these periods may have resulted in the observed exponential increase in the number of COVID-19 cases.

#### 2.3.3. Entire Data Period (1 April–31 December)

Slopes ranged from 8.15 to 43.635. The 10 states with lower slopes were Oregon (8.15), Washington (9.47), New York (9.58), the District of Columbia (11.31), Connecticut (11.89), Massachusetts (12.11), Pennsylvania (12.19), West Virginia (12.551), Virginia (12.623), and New Jersey (13.29). All these 10 states (100%) had a state-wide FC mandate by 31 December 2020. The 10 states with higher slopes were North Dakota (43.64), South Dakota (37.55), Iowa (30.10), Wisconsin (30.09), Nebraska (27.34), Utah (26.91), Tennessee (26.34), Idaho (26.12), Montana (24.95), and Arkansas (24.56). Four of these states (South Dakota, Nebraska, Tennessee, and Idaho) did not have a FC mandate by 31 December 2020. North Dakota (14 November), Iowa (17 November), and Utah (19 November) mandated FCs in the late fall. Essentially, 8 out of these 10 states (80%) did not have a FC mandate for most of the period (Supplementary Table S2).

There is a need for personal protective equipment that can effectively intervene in the chain of infection and block transmission [21]. The effects of face masks on flow resistance, aerosol filtration, and prevention of smear infection have been highlighted [21,36]. Review of data from 172 studies from around the globe indicated that with proper face coverings the risk of infection is only about 3.1% [15]. From a fluid physics point of view, flow resistance of face masks can effectively prevent transmission of SARS-CoV-2 through exhaled air [21] by asymptomatic, symptomatic, and presymptomatic individuals. The inhalation of virus-containing droplets can be prevented by using a tight-fitting FC with high filtration efficiency. In the current study, we observed that case rates were lower in states with FC mandates.

### 2.4. Correlation between Confirmed Cases and Number of Days Since Mandate

**Figure 2:**
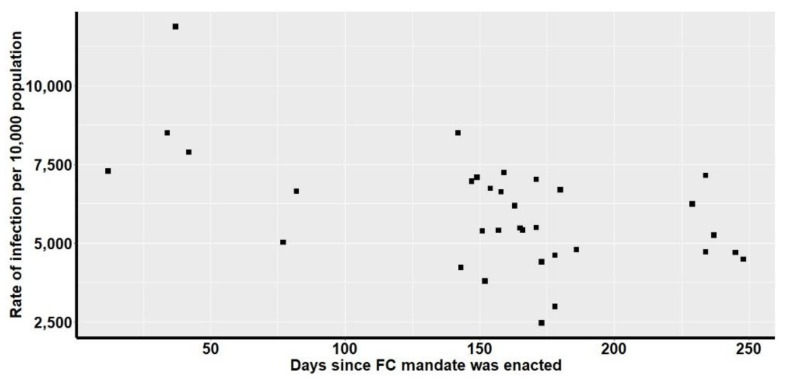
Scatter plot of correlation between the number of days since the FC mandate was passed and the rate of COVID-19 infection cases by the end of Fall 2020 (21 December).

Since wearing FCs has been linked to a reduction of risk of contracting SARS-CoV-2, it is crucial to examine how infection cases change from the time that FCs are adopted. To determine if the FC order date affects the COVID-19 case trends in 32 states with FC mandates, the correlation between infection cases per 100,000 people (*y*-axis) and the number of days since FC mandate (*x*-axis) was computed. Scatter plot representation is given in Figure 2. Results of statistical analysis revealed a negative correlation (−0.54) between the two variables. Analysis also indicated statistical significance (t = −3.5674, df = 31, *p*-value = 0.001195, 95% confidence interval = −0.7448446, −0.2407747) indicating that the correlation is not equal to 0. That is, the longer the time since passing FC mandates, the lower the rate of infection cases.

## 3. Materials and Methods

### 3.1. Data Collection

Data on confirmed COVID-19 cases were obtained from the Department of Public Health of each state (Supplementary Table S1) designated by the American National Standards Institute’s [ANSI/USPS] alphabetical codes as AL, AK, AZ, AR, CA, CO, CT, DE, DC, FL, GA, ID, IL, IN, IA, KS, KY, LA, MD, MA, MI, MN, MS, MO, MT, NE, NV, NJ, NM, NY, NC, ND, OH, OK, OR, PA, SC, SD, TN, TX, UT, WA, WI, and WY. Data on the rate of cases per 100,000 people for each state were collected from the Centers for Disease Control and Prevention (https://covid.cdc.gov/covid-data-tracker/#trends_totalandratecases, accessed on 9 February 2021). All data were collected between 10 December 2020–20 January 2021. States with fewer cumulative number of confirmed infection cases (HI, ME, NH, RI, and VT) and those that most recently mandated face coverings (VA and WV) were excluded from the analysis due to data variation resulting from continuous updates of recent daily cases. Face covering (FC) mandated states are defined as states that enacted FC mandates requiring its citizens to wear FC in public places (Supplementary Table S1).

### 3.2. Data Analysis

Statistical analysis was performed with R (v 3.5.1) programming language and Microsoft Excel 2010 for Windows. R (v 3.5.1) was used to perform Pearson correlation analysis and generate a scatter plot. Excel for Windows was used to compute regression analysis, generate charts, and construct tables. *p*-value was predetermined at <0.05 for all statistical analyses. The coefficient of determination (R^2^) for the exponential increase in cumulative confirmed cases and daily new cases was computed for data collected for summer (20 June–22 September) and fall (22 September–21 December) of 2020. R^2^ was later generated for the whole data (1 April–31 December 2020). R^2^ values for the entire data were lower because of large fluctuations in data, especially during summer–fall transitioning. The change in infection cases per 100,000 people over time was calculated using the slope of three different regression equations for summer (20 June–22 September) and fall (22 September–21 December) exponential increase in cases, and for the entire timeframe (1 April–31 December). The slope of the equation represents the rate of change in cases over a period.

### 3.3. Projection of Number of Cases Prevented by Face Covering Mandate

**Figure 3:**
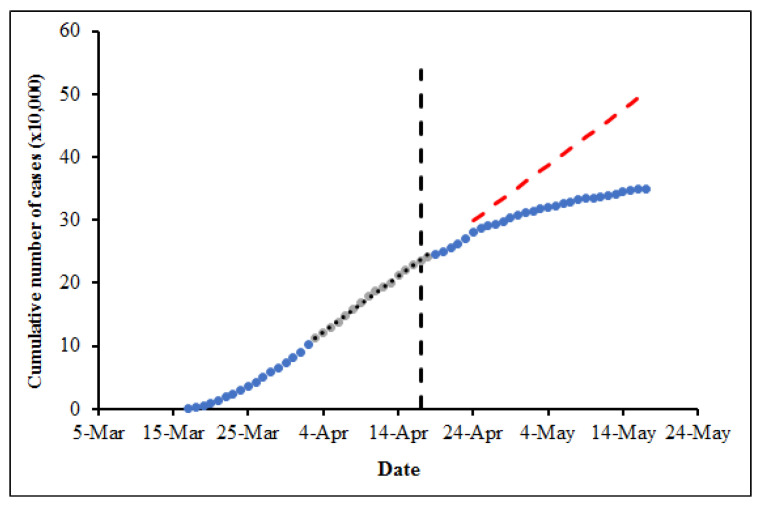
Cumulative number of COVID-19 cases (blue dotted line) between 17 March–17 May 2020 for New York state. Vertical black dash line indicates the onset of FC mandate (17 April 2020). Linear regression (black dotted line) through the data was used to predict pre-FC mandate infection data trend. The red dash line denotes projection based on linear regression of cases without FC mandate. The difference estimated the number of cases prevented by FC mandate.

Methods described by [20] were modified. The number of COVID-19 infections prevented by the use of FC was estimated by projection. Data trends before FC mandates were extrapolated, and the difference between reported and estimated cases were computed (Figure 3). The projection was performed by establishing a linear relationship between the cumulative number of cases (*y*-axis) and date (*x*-axis). The linear regression equation was generated using data from 10–20 days preceding FC mandate. Projection was restricted to 21- and 28 days post FC mandate to minimize variation and ensure reliability of the projection. The difference between the projected number of cases and actual reported cases after the FC mandate was enacted to provide the estimated number of cases prevented by the FC mandate. The seven-day period post FC mandate data was excluded from computation because the incubation period of SARS-CoV-2 is estimated to be 5 days (median) [29].

### 3.4. Correlation between the Rate of Cases and Days Since Mandate

To determine whether the date that the FC mandate was enacted impacted the COVID-19 cases, Pearson correlation analysis was performed between the number of days since the date FC was mandated (*x*-axis) vs. the number of cases per 100,000 people (*y*-axis). The statistical significance of the correlation was tested at *p* < 0.05.

## 4. Conclusions

Different mitigating measures to slow the spread of SARS-CoV-2 such as lockdowns, quarantine, required minimum distance, isolation, stay-at-home orders, and shelter-in-place have been taken with little or no debate about their effectiveness, resulting in utmost compliance. However, the use of face coverings is a highly debated measure, so its implementation varied greatly from one location to another. Physical distance rules can usually be achieved during lockdown, quarantine, shelter-in-place, and isolation. But what happens after the end of lockdown with the re-opening of businesses and gathering of people in public places? Additional effective protection is crucial to lower or stabilize infection rates.

In the current study, we analyzed COVID-19 confirmed cases from 1 April to 31 December 2020 for U.S. states with and without face covering mandates. To understand the effectiveness of face coverings during the pandemic, we analyzed the daily new infection cases, cumulative confirmed cases, and infection rate per 100,000 people for up to 46 states in the U.S. Collected data indicated an exponential increase in the number of cases during the summer and fall of 2020. Reduction in cases was, however, observed in many states that enacted a face covering mandate. The distinctive change emerged within days of passing the face covering mandate (Supplementary Figure S1). The outcome of our research illustrated that face coverings made an impact by reducing confirmed cases after mandates (Table 2 and Table 3).

To evaluate the effectiveness of face coverings beyond the 28 days included in the projection, the correlation between infection cases per 100,000 people and the number of days since the implementation of the mandate, was assessed. A statistically significant (*p* = 0.001) negative correlation (−0.54) was observed. The results highlighted the potential of community-wide wearing of face coverings in reducing the COVID-19 cases. All face coverings can offer fundamental protection if they are used properly and have sufficient flow resistance [21]. It is essential to note that if not properly worn, users will touch the face more frequently to adjust the fit of the mask which can increase the likelihood of smear infection. It is also important to emphasize that face coverings should not only cover the mouth but also create a physical transmission barrier over both the mouth and nose. Particularly, the communal use of face coverings in conjunction with other mitigating measures provides maximum protection against transmission.

## Figures and Tables

**Table 1:** States with face covering mandates and estimates from the projection of the number of cases prevented by 21 and 28 days after face covering mandates were enacted.

SN	States	Total Cases *	Effective Date	R^2^ Value	Linear Range (Date)	21 Days PD	28 Days PD
i	Alabama	399,150	16 July	0.9973	9 July–18 July	5804	10,763
ii	Arkansas	251,746	20 July	0.9985	7 July–24 July	762	1921
iii	California	2,621,277	18 June	0.9819	12 June–24 June	−74,276	−114,937
iv	Colorado	358,947	17 July	0.9977	5 July–21 July	189	1293
v	Connecticut	205,994	20 April	0.9936	12 April–24 April	5972	9135
vi	Delaware	64,475	28 April	0.9887	14 April–29 April	862	1669
vii	DC	31,457	22 July	0.9948	13 July–28 July	136	265
viii	Illinois	1,024,039	1 May	0.9979	24 April–7 May	6532	14,770
ix	Indiana	558,560	27 July	0.9973	21 July–1 August	−1828	−2624
x	Iowa	295,353	17 November	0.9900	3 November–16 November	49,750	69,448
xi	Kansas	242,322	3 July	0.9954	27 June–8 July	708	932
xii	Louisiana	341,431	11 July	0.9946	5 July–17 July	14,834	23,393
xiii	Maryland	306,674	31 July	0.9981	23 July–4 August	6241	8725
xiv	Massachusetts	417,829	1 May	0.9958	17 April–1 May	23,816	35,104
xv	Michigan	562,553	5 October	0.9903	26 September–11 October	−29,743	−64,892
xvi	Minnesota	434,413	25 July	0.9955	18 July–31 July	1017	609
xvii	Mississippi	239,082	30 September	0.9973	20 September–6 October	−5019	−6405
xviii	Montana	86,102	15 July	0.9953	7 July–18 July	−31	−140
xix	Nevada	246,309	24 June	0.9774	10 June–23 June	−16,158	−20,197
xx	New Jersey	579,182	8 July	0.9987	21 June–5 July	488	660
xxi	New Mexico	154,954	6 May	0.9969	26 April–8 May	611	671
xxii	New York	1,127,777	17 April	0.9971	3 April–18 April	111,417	160,956
xxiii	North Carolina	614,355	26 June	0.9965	17 June–30 June	−9978	−13981
xxiv	North Dakota	94,716	14 November	0.9953	4 November–17 November	13,966	21413
xxv	Ohio	770,977	23 July	0.9974	9 July–22 July	9491	11,818
xxvi	Oregon	124,476	1 July	0.9980	19 June–1 July	−877	−1080
xxvii	Pennsylvania	713,310	1 July	0.9966	22 June–7 July	−4589	−5775
xxviii	Texas	1,938,551	3 July	0.9930	26 June–9 July	−35,026	−41,723
xxix	Utah	303,723	9 November	0.9941	4 November–13 November	−1898	1385
xxx	Washington	271,595	26 June	0.9954	21 June–2 July	−2417	−2749
xxxi	Wisconsin	548,134	1 August	0.9985	20 July–2 August	3603	4212
xxxii	Wyoming	46,719	9 December	0.9944	23 November–8 December	4212	5064

Keys: * = Total number of cases documented by respective states as of 11 January 2021; PD = projected difference indicating estimated number of cases prevented by FC order by 21- or 28-day period; negative values (−) = increase in number of cases. Number of states with a reduced number of daily cases after 21 days post FC order = 20. Percentage of states where FC order reduced number of daily cases 21 days post FC order = 63%. Number of states with a reduced number of daily cases 28 days post FC order = 21. Percentage of states where FC order reduced the number of daily cases 28 days post FC order = 66%.

**Table 2:** Lowest summer and fall rates of confirmed COVID-19 infection cases.

Summer 2020					Fall 2020				
SN	States	R-LDS	M. by 9/22	M. Date	SN	States	R-LDF	M. by 12/21	M. Date
1	Oregon	742.4	Y	1 July	1	Oregon	2460	Y	1 July
2	Wyoming	866.7	N	9 December	2	Washington	2976.2	Y	26 June
3	Alaska	950	N		3	Virginia	3684.4	Y	14 December
4	Montana	1019.9	Y	15 July	4	D of Columbia	3788.9	Y	22 July
5	Washington	1092.5	Y	26 June	5	West Virginia	4092.1	Y	14 December
6	Colorado	1147	Y	17 July	6	Maryland	4224.5	Y	31 July
7	Pennsylvania	1184.6	Y	1 July	7	Pennsylvania	4402.4	Y	1 July
8	Ohio	1247.7	Y	23 July	8	New York	4480	Y	17 April
9	Michigan	1305.3	N	5 October	9	North Carolina	4611.4	Y	June
10	New Mexico	1325.3	Y	6 May	10	Connecticut	4694.6	Y	20 April

Keys: M = mandate, Y = yes, N = no, R-LDS = infection cases per 100,000 people on last day of summer 2020; R-LDF = infection cases per 100,000 people on last day of fall 2020. Most states with lowest case rates have earliest FC mandates.

**Table 3:** Highest summer and fall rates of confirmed COVID-19 infection cases.

Summer 2020					Fall 2020				
SN	States	R-LDS	M by 9/22	M. Date	SN	States	R-LDF	M by 12/21	M. Date
1	Louisiana	3511.7	Y	11 July	1	North Dakota	11,869.5	Y	14 November *
2	Mississippi	3177.7	N	30 September	2	South Dakota	10747	N	
3	Florida	3165	N		3	Iowa	8498.3	Y	14 November *
4	Georgia	3050.3	N		4	Wisconsin	8490.5	Y	1 August
5	Alabama	2989.6	Y	16 July	5	Nebraska	8121.5	N	
6	Arizona	2951.7	N		6	Utah	7884.8	Y	9 November *
7	South Carolina	2734.4	N		7	Tennessee	7754.6	N	
8	Tennessee	2711.1	N		8	Idaho	7323.6	N	
9	Iowa	2576.8	N	17 November	9	Wyoming	7277.3	Y	9 December *
10	Arkansas	2550.9	Y	20 July	10	Montana	7234.8	Y	15 July

Keys: M = mandate, Y = yes, N = no, R-LDS = infection cases per 100,000 people on last day of summer 2020; R-LDF = infection cases per 100,000 people on last day of fall 2020, * = only few days/weeks of FC mandate in 2020.

## Data Availability

Sources of all data are provided in Supplementary Table S1.

## Supplementary Materials

Our definition of face coverings is based on CDC’s guideline (https://www.cdc.gov/coronavirus/2019-ncov/prevent-getting-sick/about-face-coverings.html, accessed on 1 February 2021). Other supplementary information are presented in Supplementary Figure S1 and Supplementary Tables S1 and S2 (available online at https://www.mdpi.com/article/10.3390/ijerph18073666/s1).
