# Supplementary material for: Effectiveness of Face Coverings in Mitigating the COVID-19 Pandemic in the United States
Source: Int J Environ Res Public Health. 2021 Apr 1;18(7):3666. doi: 10.3390/ijerph18073666 (PMC8036758; doi:10.3390/ijerph18073666)
Supplement: Supplementary file 1 [file ijerph-18-03666-s001.pdf]

SUPPLEMENTARY FIGURE 1

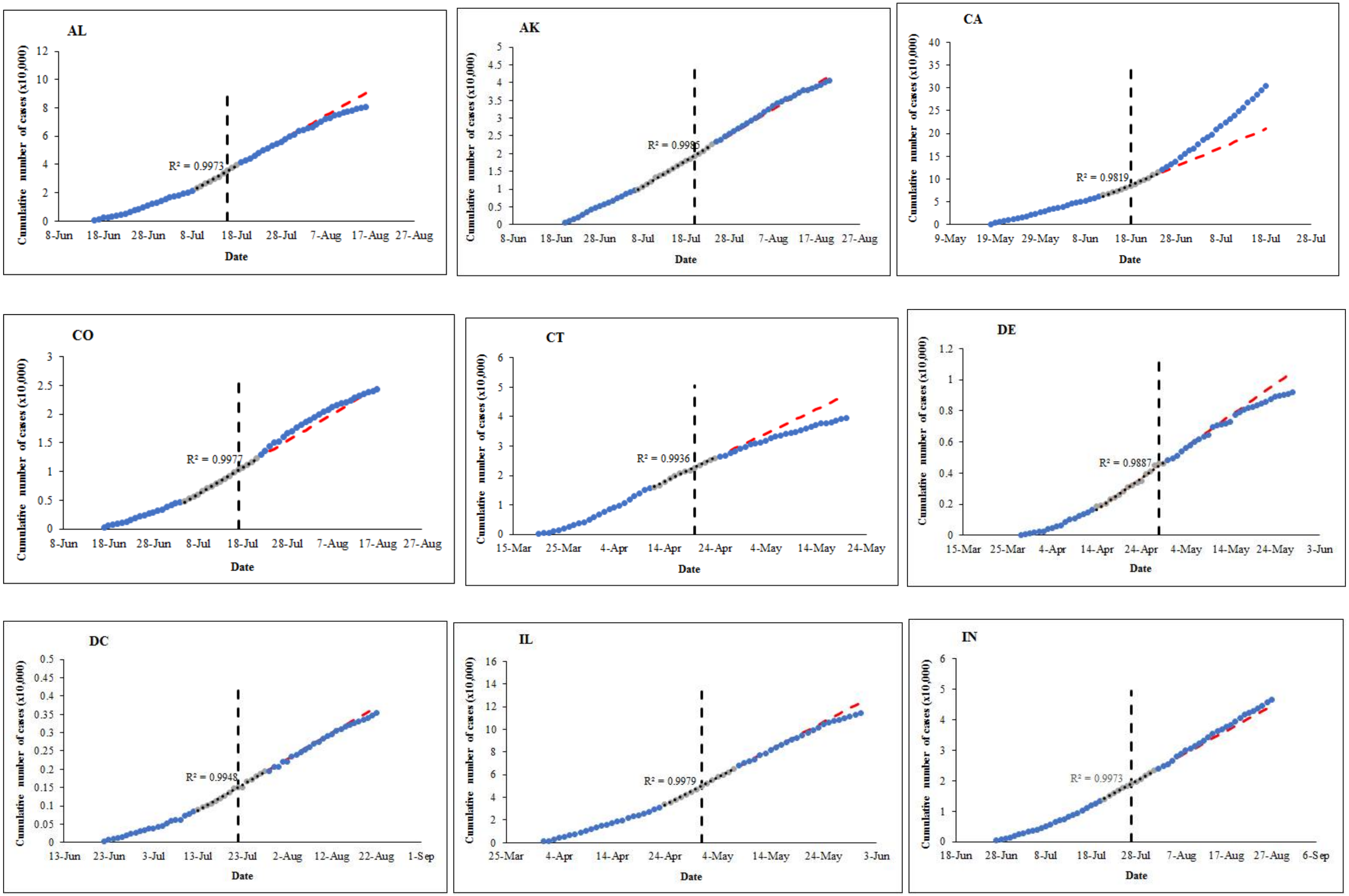

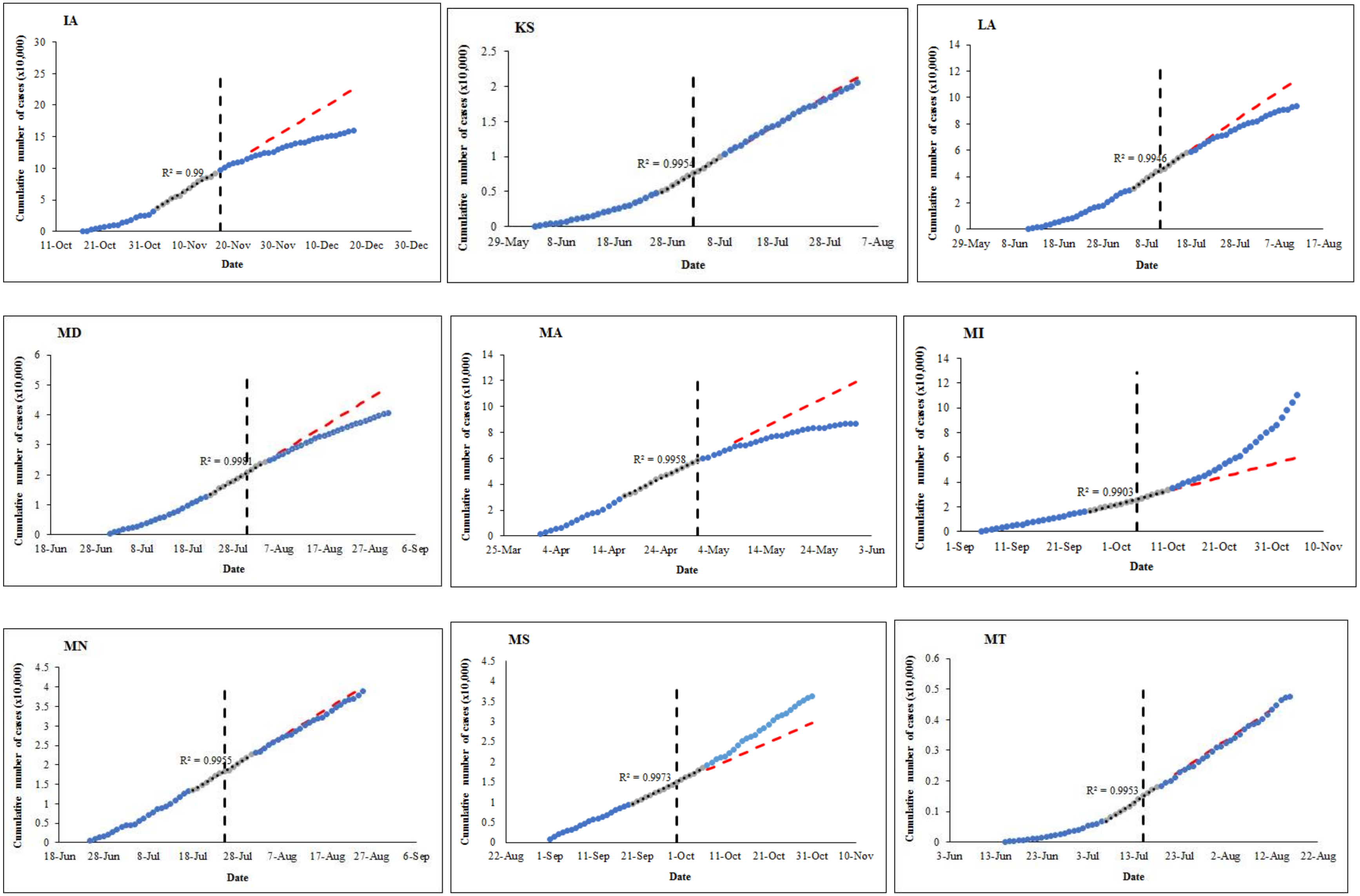
IA
R² = 0.99
KS
R² = 0.9954
LA
R² = 0.9946
MD
R² = 0.9991
MA
R² = 0.9958
MI
R² = 0.9903
MN
R² = 0.9955
MS
R² = 0.9973
MT
R² = 0.9953
Cumulative number of cases (x10,000)
Date

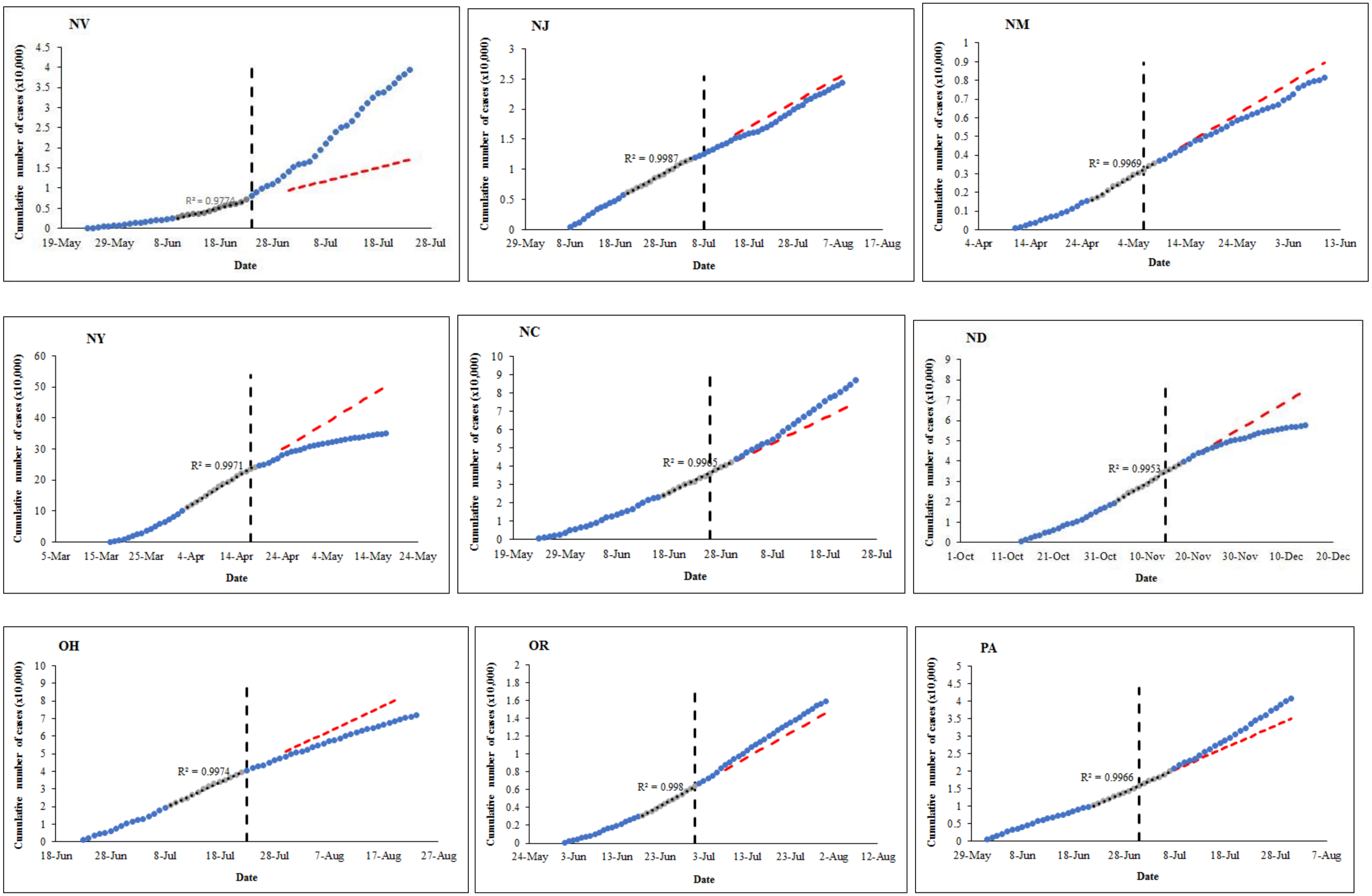

NV
R² = 0.9774
Cumulative number of cases (x10,000)
Date
19-May 29-May 8-Jun 18-Jun 28-Jun 8-Jul 18-Jul 28-Jul
NJ
R² = 0.9987
Cumulative number of cases (x10,000)
Date
29-May 8-Jun 18-Jun 28-Jun 8-Jul 18-Jul 28-Jul 7-Aug 17-Aug
NM
R² = 0.9969
Cumulative number of cases (x10,000)
Date
4-Apr 14-Apr 24-Apr 4-May 14-May 24-May 3-Jun 13-Jun
NY
R² = 0.9971
Cumulative number of cases (x10,000)
Date
5-Mar 15-Mar 25-Mar 4-Apr 14-Apr 24-Apr 4-May 14-May 24-May
NC
R² = 0.9965
Cumulative number of cases (x10,000)
Date
19-May 29-May 8-Jun 18-Jun 28-Jun 8-Jul 18-Jul 28-Jul
ND
R² = 0.9953
Cumulative number of cases (x10,000)
Date
1-Oct 11-Oct 21-Oct 31-Oct 10-Nov 20-Nov 30-Nov 10-Dec 20-Dec
OH
R² = 0.9974
Cumulative number of cases (x10,000)
Date
18-Jun 28-Jun 8-Jul 18-Jul 28-Jul 7-Aug 17-Aug 27-Aug
OR
R² = 0.998
Cumulative number of cases (x10,000)
Date
24-May 3-Jun 13-Jun 23-Jun 3-Jul 13-Jul 23-Jul 2-Aug 12-Aug
PA
R² = 0.9966
Cumulative number of cases (x10,000)
Date
29-May 8-Jun 18-Jun 28-Jun 8-Jul 18-Jul 28-Jul 7-Aug

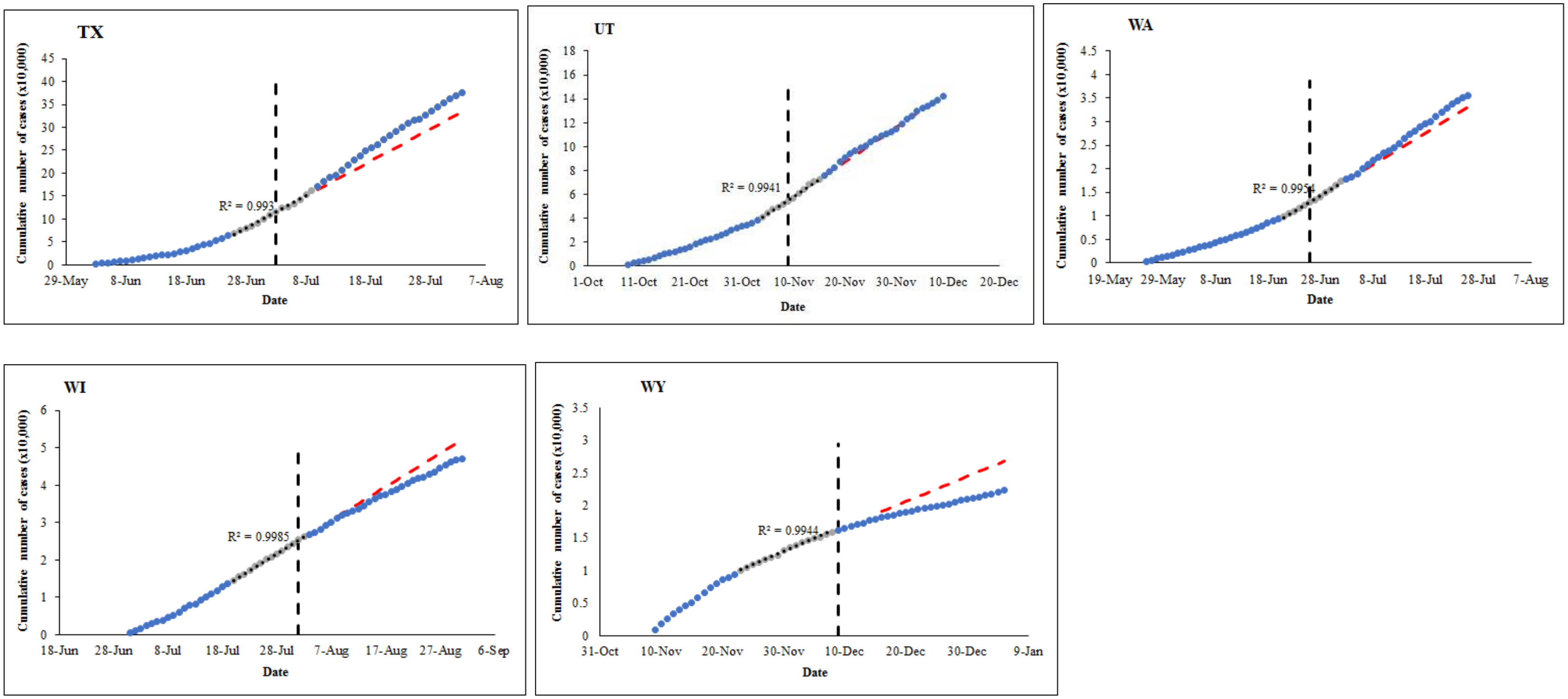

Supplementary Figure S1: Cumulative number of COVID-19 cases (y-axis) in U.S. states over time (x-axis). The chart is showing trend of cases (blue dotted line) for one month pre- and post- FC mandate (total = 2 months) for each state with face coverings mandate. The black vertical line is the date when face coverings order was passed. The gray segment through the case data was used to derive regression equation (black dotted line) that was used to project the number of cases (red line) that would have been recorded if FC order were not passed. The prediction was limited to 28 days post FC order to limit variation. Seven days post FC mandate was omitted from the prediction because the incubation period of COVID-19 is estimated to be 5 days.

Supplementary Table S1: U.S. states with and without state-wide order mandating the wearing of face coverings in public places. Data on confirmed COVID-19 cases were collected from or through the websites of the Department of Public Health of each state.

State/Code	State-wide order	Effective date	Sources – daily cases and cumulative infection cases data	Face covering mandates
Alabama [AL]	Yes	July 16	https://www.alabamapublichealth.gov/covid19/index.html	https://governor.alabama.gov/assets/2020/12/Safer-at-Home-Order-Final-12.9.2020.pdf
Alaska [AK]	No			
Arizona [AZ]	No			
Arkansas [AR]	Yes	July 20	https://www.healthy.arkansas.gov/programs-services/topics/novel-coronavirus	https://governor.arkansas.gov/images/uploads/executiveOrders/EO_20-43.pdf
California [CA]	Yes	June 18	https://covid19.ca.gov/state-dashboard/	https://www.cdph.ca.gov/Programs/CID/DCDC/CDPH%20Document%20Library/COVID-19/Guidance-for-Face-Coverings_06-18-2020.pdf
Colorado [CO]	Yes	July 17	https://covid19.colorado.gov/data	https://www.colorado.gov/governor/sites/default/files/inline-files/D%202020%20138%20Mask%20Order.pdf
Connecticut [CT]	Yes	April 20	https://data.ct.gov/Health-and-Human-Services/COVID-19-daily-and-cumulative-cases-hospitalizatio/5dch-cm68	https://portal.ct.gov/-/media/Office-of-the-Governor/Executive-Orders/Lamont-Executive-Orders/Executive-Order-No-7BB.pdf?la=en
Delaware [DE]	Yes	April 28	https://myhealthycommunity.dhss.delaware.gov/locations/state	https://governor.delaware.gov/health-soe/twenty-seventh-modification-state-of-emergency-declaration/<br>https://governor.delaware.gov/health-soe/thirteenth-state-of-emergency/<br>https://coronavirus.delaware.gov/guidance-for-face-coverings/
DC	Yes	July 22	https://coronavirus.dc.gov/data	https://coronavirus.dc.gov/maskorder
Florida [FL]	No			
Georgia [A]	No			
Hawaii [HI]	Yes		-	https://governor.hawaii.gov/wp-content/uploads/2020/10/2010095-ATG_Fourteenth-Proclamation-for-COVID-19-distribution-signed.pdf<br>https://governor.hawaii.gov/wp-content/uploads/2020/11/2011051-ATG_Fifteenth-Proclamation-Related-to-the-COVID-19-Emergency-distribution-signed.pdf
Idaho [ID]	No			
Illinois [IL]	Yes	May 1	https://www.dph.illinois.gov/covid19/covid19-statistics	https://www.dph.illinois.gov/covid19/community-guidance/mask-use<br>https://www2.illinois.gov/Pages/Executive-Orders/ExecutiveOrder2020-32.aspx
Indiana [IN]	Yes	July 27	https://www.coronavirus.in.gov/2393.htm	https://www.in.gov/gov/files/Executive_Order_20-48_Color-Coded_County_Assessments.pdf
Iowa [IA]	Yes	Nov 17	https://coronavirus.iowa.gov/pages/case-counts	https://governor.iowa.gov/sites/default/files/documents/Public%20Health%20Proclamation%20-%202020.12.09.pdf
Kansas [KS]	Yes	Jul 3	https://www.coronavirus.kdheks.gov/160/COVID-19-in-Kansas	https://governor.kansas.gov/wp-content/uploads/2020/11/EO-20-68-Face-Coverings-protocol-Executed-1.pdf<br>https://governor.kansas.gov/wp-content/uploads/2020/07/20200702093130003.pdf
Kentucky [KY]	Yes	July 9	-	https://governor.ky.gov/attachments/20201203_Executive-Order_2020-996_Face-coverings.pdf

Louisiana [LA]	Yes	July 11	https://ldh.la.gov/Coronavirus/	https://gov.louisiana.gov/assets/Proclamations/2020/168-JBE-2020-State-of-Emergency-Renewing-COVID-19.pdf
Maine [ME]	Yes	May 1	-	https://www.maine.gov/governor/mills/sites/maine.gov.governor.mills/files/inline-files/An%20Order%20to%20Revise%20Indoor%20Gathering%20Limits%2C%20Strengthen%20Face%20Covering%20Requirements%20and%20Delegate%20Certain%20Authority.pdf
Maryland [MD]	Yes	July 31	https://coronavirus.maryland.gov/	https://governor.maryland.gov/wp-content/uploads/2020/07/Gatherings-10th-AMENDED-7.29.20.pdf
Massachusetts [MA]	Yes	May	https://www.mass.gov/info-details/covid-19-response-reporting#covid-19-interactive-data-dashboard-	https://www.mass.gov/doc/covid-19-order-55/download
Michigan [MI]	Yes	Oct 5	https://www.michigan.gov/coronavirus/0,9753,7-406-98163_98173---,00.html	https://www.michigan.gov/coronavirus/0,9753,7-406-98178_98455-543708--,00.html
Minnesota [MN]	Yes	July 25	https://www.health.state.mn.us/diseases/coronavirus/situation.html#cases1	https://www.leg.mn.gov/archive/execorders/20-81.pdf
Mississippi [MS]	Yes	Sep 30	https://msdh.ms.gov/msdhsite/_static/14,21882,420,873.html	https://www.sos.ms.gov/content/executiveorders/ExecutiveOrders/1536.pdf
Missouri [MO]	No			
Montana [MT]	Yes	July 15	https://covid19.mt.gov/	https://covid19.mt.gov/_docs/2020-11-17_Directive-on-Group-Size-and-Capacity-FINAL.pdf
Nebraska [NE]	No			
Nevada [NV]	Yes	June 24	https://nvhealthresponse.nv.gov/#covid-data-tracker	https://nvhealthresponse.nv.gov/wp-content/uploads/2020/06/Directive-024-Face-Coverings.pdf
New Hampshire [NH]	Yes	Nov 20	-	https://www.governor.nh.gov/sites/g/files/ehbemt336/files/documents/emergency-order-74.pdf
New Jersey [NJ]	Yes	July 8	https://covid19.nj.gov/	https://nj.gov/infobank/eo/056murphy/pdf/EO-163.pdf
New Mexico [NM]	Yes	May 6	https://cvprovider.nmhealth.org/public-dashboard.html	https://www.governor.state.nm.us/wp-content/uploads/2020/05/05_15_2020_PHO.pdf
New York [NY]	Yes	April 17	https://coronavirus.health.ny.gov/home	https://www.governor.ny.gov/news/no-20217-continuing-temporary-suspension-and-modification-laws-relating-disaster-emergency
North Carolina [NC]	Yes	June	https://covid19.ncdhhs.gov/dashboard/cases	https://files.nc.gov/governor/documents/files/EO181-Modified-Stay-at-Home-Early-Closure-Order.pdf
North Dakota [ND]	Yes	Nov 14	https://www.health.nd.gov/diseases-conditions/coronavirus/north-dakota-coronavirus-cases	https://www.governor.nd.gov/sites/www/files/documents/executive-orders/SHO%20Order%202020-08%20Signed.pdf
Ohio [OH]	Yes	July 23	https://coronavirus.ohio.gov/wps/portal/gov/covid-19/dashboards/overview/	https://governor.ohio.gov/wps/portal/gov/governor/media/news-and-media/covid19-update-07222020
Oklahoma [OK]	No			
Oregon [OR]	Yes	July 1	https://coronavirus.oregon.gov/Pages/default.aspx	https://govstatus.egov.com/or-oha-face-coverings
Pennsylvania [PA]	Yes	July 1	https://www.health.pa.gov/topics/disease/coronavirus/Pages/Cases.aspx	https://www.health.pa.gov/topics/Documents/Diseases%20and%20Conditions/Updated%20Order%20of%20the%20Secretary%20Requiring%20Universal%20Face%20Coverings.pdf

South Carolina [SC]	No			
South Dakota [SD]	No			
Tennessee [TN]	No			
Texas [TX]	Yes	July 3	https://www.dshs.state.tx.us/coronavirus/	https://open.texas.gov/uploads/files/organization/opentexas/EO-GA-29-use-of-face-coverings-during-COVID-19-IMAGE-07-02-2020.pdf
Utah [UT]	Yes	Nov 9	https://coronavirus.utah.gov/case-counts/	https://coronavirus-download.utah.gov/Health/UPHO-2020-26-Updated-Statewide-COVID-19-Restrictions.pdf
Vermont [VT]	Yes	Aug 1	-	https://governor.vermont.gov/sites/scott/files/documents/ADDENDUM%202%20TO%20AMENDED%20AND%20RESTATED%20EXECUTIVE%20ORDER%20NO.%2001-20.pdf
Virginia [VA]	Yes	Dec 14	-	https://www.governor.virginia.gov/media/governorvirginiagov/executive-actions/EO-72-and-Order-of-Public-Health-Emergency-Nine-Common-Sense-Surge-Restrictions-Certain-Temporary-Restrictions-Due-to-Novel-Coronavirus-[COVID-19].pdf
Washington [WA]	Yes	June 26	https://www.doh.wa.gov/Emergencies/COVID19/DataDashboard	https://www.doh.wa.gov/Portals/1/Documents/1600/coronavirus/ClothFacemasks.pdf
West Virginia [WV]	Yes!	Dec 14	-	https://governor.wv.gov/Pages/Statewide-Indoor-Face-Covering-Requirement.aspx
Wisconsin [WI]	Yes	Aug 1	https://www.dhs.wisconsin.gov/covid-19/cases.htm	https://content.govdelivery.com/attachments/WIGOV/2020/11/20/file_attachments/1607585/EmO01-NovFaceCovering.pdf
Wyoming [WY]	Yes	Dec 9	https://health.wyo.gov/publichealth/infectious-disease-epidemiology-unit/disease/novel-coronavirus/covid-19-map-and-statistics/	https://health.wyo.gov/wp-content/uploads/2020/12/Order4_StatewideFaceCoveringOrder_Dec72020.pdf

Supplementary Table S2: Change in trends of cases rate per 100,000 people during summer and fall exponential increase in daily cases and confirmed cumulative cases. Higher slopes indicate higher case rate and lower slopes indicate lower case rates.

SN	States	All data		Summer surge data			Fall surge data		
		R^2	Slope (d^{-1})	R^2	Slope (d^{-1})	R-LDS	R^2	Slope (d^{-1})	R-LDF
1	Alabama	0.9425	24.555	0.9899	27.364	2989.6	0.9495	38.124	6624.9
2	Alaska[a]	0.7162	18.897	0.9912	9.769	950	0.9546	58.777	5818.2
3	Arizona[a]	0.9272	22.187	0.8841	23.349	2951.7	0.8550	34.016	6338.3
4	Arkansas	0.9090	24.652	0.9982	21.886	2550.9	0.9679	45.416	6730.3
5	California	0.8926	15.839	0.9840	18.027	1985	0.8129	24.569	4789.3
6	Colorado	0.7188	16.308	0.9911	6.774	1147	0.9286	51.867	5401.1
7	Connecticut	0.7403	11.886	0.9901	3.0394	1575.2	0.8973	34.373	4694.6
8	Delaware	0.8622	15.653	0.9970	9.8417	2029.3	0.881	33.07	5243.2
9	D of Columbia	0.9470	11.307	0.9945	8.2233	2128.4	0.9148	17.668	3788.9
10	Florida[a]	0.9632	21.976	0.9471	31.396	3165	0.9417	25.567	5552.1
11	Georgia[a]	0.9650	21.386	0.9901	28.567	3050.3	0.9402	25.815	5570.4
12	Idaho[a]	0.8493	26.123	0.9867	22.087	2145.8	0.9752	60.193	7323.6
13	Illinois	0.8210	23.058	0.9801	12.537	2205.4	0.9557	60.820	7142.4
14	Indiana	0.7628	21.664	0.9904	11.899	1672.9	0.9357	61.306	6954.9
15	Iowa	0.8372	30.095	0.9815	18.768	2576.8	0.9607	75.765	8498.3
16	Kansas	0.8041	23.468	0.9939	15.641	1852.2	0.9471	60.535	7022.9
17	Kentucky	0.8032	18.262	0.9887	12.353	1404.1	0.9540	46.041	5468.1
18	Louisiana	0.967	21.970	0.9605	28.590	3511.7	0.9119	28.563	6179.3
19	Maryland	0.9365	13.659	0.9942	10.871	2006.3	0.9197	24.415	4224.5
20	Massachusetts	0.8135	12.112	0.9943	4.6683	1969.7	0.886	28.307	4711.6
21	Michigan	0.7486	15.042	0.9947	6.9972	1305.3	0.9328	45.611	5017.7
22	Minnesota	0.7659	23.052	0.9958	11.465	1633.1	0.9339	68.138	7080.4
23	Mississippi	0.9538	24.551	0.9862	28.606	3177.7	0.9491	36.579	6642.5
24	Missouri[a]	0.8466	21.663	0.9838	17.752	1879.7	0.9718	49.097	6001.1
25	Montana	0.7211	24.952	0.9878	10.226	1019.9	0.9857	76.922	7234.8
26	Nebraska[a]	0.8081	27.335	0.9912	13.309	2160.1	0.9652	74.254	8121.5
27	Nevada	0.8922	23.159	0.9715	24.331	2483.6	0.9183	45.909	6684.2
28	New Jersey	0.7932	13.291	0.9978	4.2512	2310.4	0.9115	34.738	5406.5
29	New Mexico	0.7427	19.359	0.9699	9.3487	1325.3	0.9289	58.788	6238.4
30	New York	0.8051	9.5751	0.9994	3.4079	2391.6	0.8734	21.314	4480.0

31	North Carolina	0.9295	16.559	0.9967	15.627	1904.1	0.9532	28.108	4611.4
32	North Dakota	0.7695	43.635	0.9334	20.923	2490.7	0.9821	122.19	11869.5
33	Ohio	0.7456	16.245	0.9979	9.6734	1247.7	0.8978	45.863	5384.1
34	Oklahoma[a]	0.8536	23.914	0.9926	21.371	2206.4	0.9512	49.480	6883.5
35	Oregon	0.8363	8.1504	0.9932	6.5497	742.4	0.9214	19.036	2460
36	Pennsylvania	0.7363	12.191	0.9989	5.9357	1184.6	0.8753	33.771	4402.4
37	South Carolina[a]	0.9627	20.628	0.9768	24.303	2734.4	0.9516	26.540	5355.4
38	South Dakota[a]	0.7611	37.554	0.8996	14.395	2169.1	0.9859	108.2	10747
39	Tennessee[a]	0.8826	26.335	0.9931	25.286	2711.1	0.9369	50.819	7754.6
40	Texas	0.939	20.858	0.9823	23.242	2470.0	0.9629	34.542	5490.3
41	Utah	0.8191	26.914	0.9906	14.972	2028.8	0.9685	68.465	7884.8
42	Virginia	0.9387	12.623	0.9966	11.185	1670.5	0.9359	20.928	3684.4
43	Washington	0.8727	9.468	0.9856	8.2791	1092.5	0.9046	20.046	2976.2
44	West Virginia	0.7151	12.551	0.9892	7.0574	802.6	0.9110	35.433	4092.1
45	Wisconsin	0.7919	30.087	0.9909	14.47	1894.9	0.9849	81.488	8490.5
46	Wyoming	0.6671	23.171	0.9959	6.7687	866.7	0.9542	81.135	7277.3

Keys: a = states without face coverings mandate in public; d = rate of change in confirmed cases per day; R-LDS = case rate per 100,000 people on last day of summer 2020 (September 22); R-LDF = case rate per 100,000 people on last day of fall 2020 (December 21).
